# Harnessing Large-Scale Herbarium Image Datasets Through Representation Learning

**DOI:** 10.3389/fpls.2021.806407

**Published:** 2022-01-13

**Authors:** Barnaby E. Walker, Allan Tucker, Nicky Nicolson

**Affiliations:** ^1^Royal Botanic Gardens, Kew, Richmond, United Kingdom; ^2^Department of Computer Science, Brunel University London, Uxbridge, United Kingdom

**Keywords:** deep learning, digitized herbarium specimens, natural history collections, machine learning, computer vision

## Abstract

The mobilization of large-scale datasets of specimen images and metadata through herbarium digitization provide a rich environment for the application and development of machine learning techniques. However, limited access to computational resources and uneven progress in digitization, especially for small herbaria, still present barriers to the wide adoption of these new technologies. Using deep learning to extract representations of herbarium specimens useful for a wide variety of applications, so-called “representation learning,” could help remove these barriers. Despite its recent popularity for camera trap and natural world images, representation learning is not yet as popular for herbarium specimen images. We investigated the potential of representation learning with specimen images by building three neural networks using a publicly available dataset of over 2 million specimen images spanning multiple continents and institutions. We compared the extracted representations and tested their performance in application tasks relevant to research carried out with herbarium specimens. We found a triplet network, a type of neural network that learns distances between images, produced representations that transferred the best across all applications investigated. Our results demonstrate that it is possible to learn representations of specimen images useful in different applications, and we identify some further steps that we believe are necessary for representation learning to harness the rich information held in the worlds’ herbaria.

## Introduction

Herbarium collections provide primary data for scientific activities across plant science (Page et al., 2015; Meineke et al., 2019). The push toward digitizing collections has made this data more widely available and has increasingly enabled studies encompassing larger groups of species and a greater proportion of the world. However, current digitization workflows focus on capturing information from specimen labels, leaving a wealth of data in hard-to-browse specimen images. New technologies, like deep learning, can help researchers make full use of this rich data source (Pearson et al., 2020; Orr et al., 2021).

### Digitized Herbarium Specimens

A herbarium specimen is a physical record of an individual plant, providing verifiable data for reproducible science (Nic Lughadha et al., 2019). Pieces of a plant are dried, pressed, and mounted to display the specimen’s features clearly. Information about the collection event is recorded on a specimen label, including who collected it, when, and where. However, this collection metadata is often incompletely recorded or not recorded at all, especially for older specimens. Taxonomic determinations are recorded on the specimen sheet and may comprise multiple revisions long after the specimen was collected. During digitization, both images of specimens and the details on specimen labels are captured.

The past two decades have seen initiatives for digitizing natural history collections established at institutional, national, and international levels (Nelson and Ellis, 2019). These initiatives have mobilized a vast amount of biodiversity data held in the estimated 396 million specimens across 3,500 herbaria (Thiers, 2021), making it available to researchers through aggregated portals like GBIF (2021) and iDigBio (2021), and virtual herbaria like the Reflora Virtual Herbarium (Reflora - Virtual Herbarium, 2021). The increasing pace of digitization and data mobilization enables more and newer studies each year; in 2019, more than two studies a day cited data published through GBIF (Paton et al., 2020).

Despite this progress, specimen data is not entirely digitally mobilized. Variation in approaches to digitization projects (Dillen et al., 2019) means different amounts of data are captured and made available; images may be available with minimal metadata, full metadata may be available with no image, or anything in between. As a result, while digital aggregators hold tens and hundreds of millions of records from preserved specimens, only two-fifths of those in GBIF (2021) and one-half of those in iDigBio (2021) have images available. This situation is only exacerbated for smaller herbaria not part of well-funded digitization projects, which undoubtedly contain many important specimens for local flora (Marsico et al., 2020).

### Deep Learning

Deep learning, a branch of machine learning that uses neural network models composed of many stacked layers, is particularly effective for image-based tasks. The stacked network layers can learn patterns from images that correspond to details like edges, circles, or even eyes—features that would need to be manually extracted for other machine learning or modeling techniques (LeCun et al., 2015). Recent studies demonstrate good performance for tasks that populate or enrich collection metadata from digitized specimen images, including species identification (Wäldchen and Mäder, 2018; Little et al., 2020), masking specimen labels (White et al., 2020), extracting traits (Mirnezami et al., 2020), and classifying the phenological state of a specimen (Lorieul et al., 2019).

One disadvantage of deep learning is its data-hungry nature, usually requiring numerous carefully curated and labeled images to train models that achieve good performance. This problem is especially acute for fine-grained classification problems, like species identification, that have many potential classes with only a few images available for training. Similarly, it can take a lot of time and expertise to label a training dataset for more complex tasks like counting the number and type of organs on a specimen. This problem is exacerbated by the fact that some herbaria have so far digitized only a fraction of their collections, limiting the number of specimens available for some plant groups.

### Representation Learning

**FIGURE 1:**
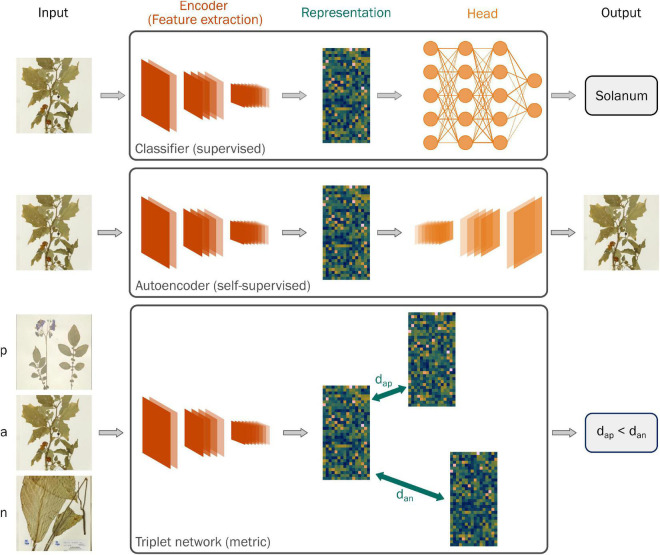
Schematic diagrams of the three types of neural networks used to learn representations of specimen images in this study: a classifier trained to predict genus, an autoencoder, and a triplet network. The schematic demonstrates the different parts of a neural network: the encoder, or feature extraction layers, that produces a lower-dimensional representation of the input and the head, which uses the representation to perform a task like predicting the genus (classifier) or reconstructing the original image (autoencoder). Representation learning focuses on training neural networks to extract features from images that transfer across different tasks. Unlike the other two networks, the triplet network takes three images as input: an anchor (a), a positive example (p) from the same class as the anchor, and a negative example (n) from a different class to the anchor. The network learns features that minimize the distance between examples from the same class (d_ap_) and maximize the distance between examples from different classes (d_an_). Inset specimen images © RBG Kew.

Deep neural networks can be viewed as being composed of two parts—an encoder network that automatically extracts features from an input and a head that uses those features to perform a particular task (Figure 1). Visual representation learning focuses on training neural networks to extract features from images that transfer across different tasks (Bengio et al., 2014). Learning generalized representations from readily available benchmarking datasets like ImageNet reduces the need to assemble large sets of labeled images for each problem.

Two popular methods for learning these representations differ in the type of task used to train the neural network. In supervised learning, neural networks are trained to classify images using a labeled dataset. Self-supervised networks, on the other hand, are trained to perform tasks where the labels are created from the images themselves, such as reconstructing the original image after some transformation or identifying which of two images is a transformed version of a target. Although supervised models have historically performed better, self-supervised models have recently achieved comparable performance (Chen et al., 2020b).

Self-supervised representation learning has gained popularity with researchers that use wildlife images from community platforms and camera traps. Both these sources produce a high volume of images with few or uncertain labels, and assembling high-quality labeled datasets presents a bottleneck for training models to mine these images for novel information. Recent work by Van Horn et al. (2021) has demonstrated the need for domain-specific datasets for learning effective representations.

Unlike natural world images, representation learning has had little attention in the realm of natural history specimens. Two recent examples include using representations learned by classifying the genera of fern images to explore morphological diversity (White et al., 2019) and using a triplet network to test evolutionary hypotheses about mimicry in butterflies (Hoyal Cuthill et al., 2019).

Efforts from herbaria like the New York Botanical Garden (NYBG) to provide datasets of herbarium specimens for species identification challenges (Little et al., 2020; de Lutio et al., 2021) offer ideal training sets for both supervised and self-supervised representation learning. However, unlike images of plants in the wild, digitized herbarium specimens are almost always accompanied by some determination of the taxon’s identity. Therefore, there may be little benefit to using self-supervision to learn representations of herbarium specimens.

Self-supervision may, however, avoid potential problems caused by the estimated large number of mislabeled specimens. Similarly, self-supervision may handle the long-tail of species represented by very few specimens (Enquist et al., 2019).

An alternative to both these methods is metric learning, where a neural network is trained to learn a distance function between different classes of images. In a triplet network (Figure 1), one implementation of metric learning (Hoffer and Ailon, 2018), a neural network is presented with three images: an anchor, a positive example from the same class as the anchor, and a negative example from a different class than the anchor. During training, the triplet network learns representations that minimize the distance between images in the same class while maximizing the distance between images in different classes. As such, metric learning offers a balance between self-supervised and supervised neural networks.

### Herbarium Specimen Representations

Here, we evaluate the potential for using a publicly available dataset of herbarium specimen images to learn generalizable representations. To do this, we train three different neural networks that serve as a progression from self-supervised to supervised learning: an autoencoder, a triplet network that selects training examples based on the specimen’s genus, and a classifier trained to predict the genus of a specimen. We explore the differences between these representations using visualizations and evaluate their potential generalizability using three downstream tasks relevant to work in herbaria.

## Materials and Methods

### Learning Representations

#### Data

We used images from the Herbarium 2021 “Half-Earth Challenge” (de Lutio et al., 2021) of the Eighth Fine-Grained Visual Categorization workshop (FGVC8) for training neural networks to learn generalized representations. The “Half-Earth” dataset—so named because it covers half of the world’s continents—contains over 2 million images of specimens collected from 5 herbaria, representing nearly 65,000 species of vascular plants across the Americas, Oceania, and the Pacific.

**FIGURE 2:**
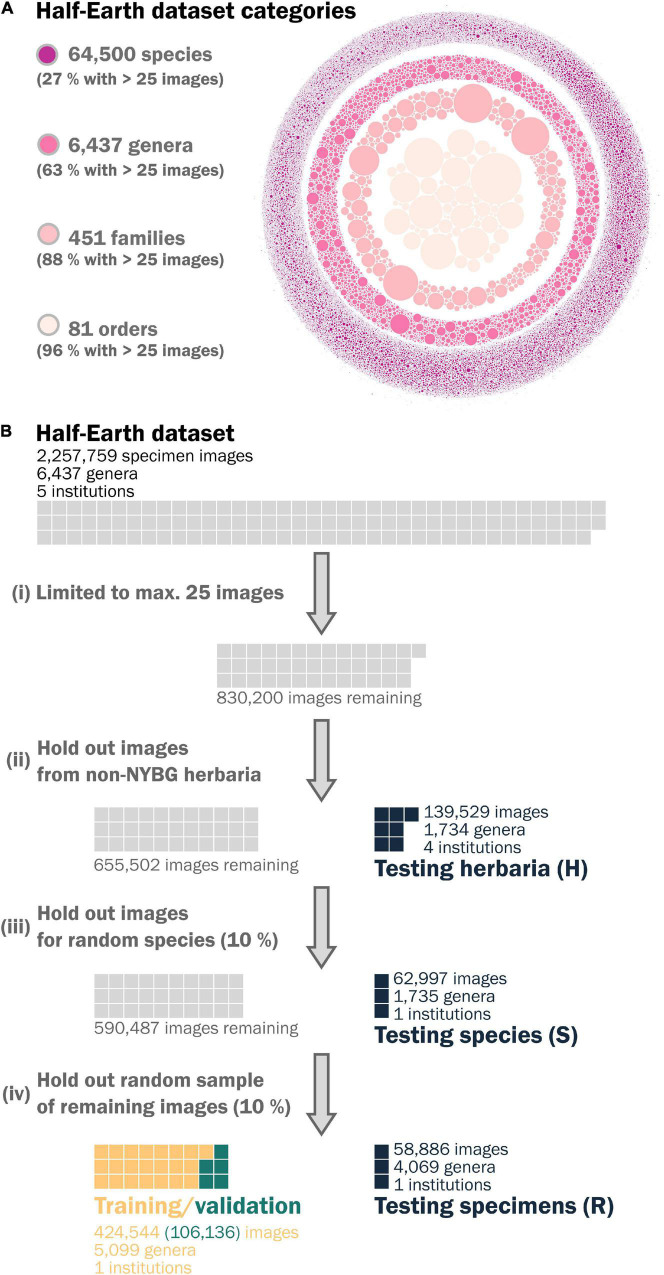
**(A)** A visual representation of the *Herbarium 2021: Half-Earth Challenge* dataset, released as part of the FGVC8 workshop illustrating the distribution of images within categories of the taxonomic hierarchy, where each dot represents the relative number of images associated with a category. **(B)** A schematic diagram of the steps we took to split the Half-Earth dataset into the training and test sets used in this study, highlighting their relative sizes.

The challenge provides a labeled set of images for participants to develop, train, and validate their models and an unlabeled set to assess their performance in the competition. We used the labeled dataset for our study, which comprises 2,257,759 images covering 64,500 species in 6,437 genera across 451 families and 81 orders of vascular plants (Figure 2A).

The Half-Earth dataset reproduces the empirical long-tailed distribution for plant species observations (Enquist et al., 2019); almost three-quarters of species have fewer than 25 images, and nearly a third have fewer than 5. To avoid over-representing a small number of species and reduce neural network training times, we downsampled the dataset to limit species to at most 25 images. We randomly sampled 25 images for species that exceeded this limit.

We subsequently split this labeled dataset sequentially to give 3 distinct test sets (Figure 2B):

1. All images from herbaria other than NYBG, by far the most represented herbarium in the dataset (139,529 images). It may seem natural for an institution to train a neural network on only their digitized images, but specimens relevant to a particular study may be distributed across herbaria. We chose this test set to examine how well representations learned from one institution will transfer to others. Hereafter referred to as test set **H**.
2. All images for a random sample of 10% of the remaining species (62,997 images). New species are still regularly discovered in the wild, and continuing digitization of a collection makes images available for species that were not used to learn the representations. We chose this test set to examine how well representations generalize to unseen species. Hereafter referred to as test set **S**.
3. A random sample of 10% of the remaining specimens (58,886 images). Herbarium collections hold multiple specimens for many taxa, but time and funding constraints mean they may focus digitization on a subset of these, such as only type specimens. We chose this test to examine how well representations learned from a set of species generalize to unseen specimens from those species. Hereafter referred to as test set **R**.

We used the remaining specimens to train the neural networks (424,544 images), reserving 20% for validation (106,136 images) during model development (Table 2). We removed images from the 3 test sets that represented genera not present in the training data to prevent the need to make predictions for classes present in the training data but missing in the test data when testing the classification network.

**TABLE 1:** Description of the three neural networks used for representation learning.

Model	Description	Pre-training dataset	Loss function
Autoencoder	A symmetric autoencoder with a ResNet-18 encoder, a latent space of 256 units, and a ResNet-18 decoder where convolutions have been replaced by resizing convolutions.	CIFAR-10	Mean squared error (MSE) of the reconstructed image.
Triplet network	A ResNet-18 encoder, through which three images are passed—an anchor, an example from the same class (positive), and an example from a different class (negative).	ImageNet	Triplet loss
Classifier	A ResNet-18 encoder with a classification head comprising two densely connected layers, each preceded by batch normalization and dropout layers.	ImageNet	Cross-entropy loss

**TABLE 2:** Description of image datasets used.

Stage	Task	Name	Source	Number of specimens
Learning representations	-	Training set	Half-earth dataset	424,544
Learning representations	-	Validation set	Half-earth dataset	106,136
Applying representations	Taxonomic identification	Herbarium test set (H)	Half-earth dataset	139,529
Applying representations	Taxonomic identification	Species test set (S)	Half-earth dataset	62,997
Applying representations	Taxonomic identification	Random specimen test set (R)	Half-earth dataset	58,886
Applying representations	Genus discrimination/Identifying mislabels	*Syzygium*	RBG, Kew	1,996
Applying representations	Genus discrimination/Identifying mislabels	*Eugenia*	RBG, Kew	8,358
Applying representations	Genus discrimination/Identifying mislabels	*Dendrobium*	RBG, Kew	1,004

We pre-sized images to 526 × 526 pixels before applying standard random transformations (flipping, rotating, zooming, warping, and brightening) during training and resizing to the final size of 256 × 256 pixels for both training and evaluation.

#### Neural Networks

All neural networks used a pre-trained ResNet-18 architecture as an encoder, producing feature representations of 512 units. We trained three different neural networks to compare the representations resulting from self-supervised learning, supervised metric learning, and supervised classification (Figure 1 and Table 1).

We chose to use the specimen images’ genus as the target for classification and for selecting positive and negative examples for the triplet network. We felt this provided a good balance between reducing the total number of classes and minimizing the variation within each class.

We trained all networks for 25 epochs on a Tesla V100 GPU and measured the performance of the final models on the 3 test sets described above.

#### Comparing Representations

We compared the separability of taxonomic groups in the extracted representations using silhouette scores, a measure of the average distance between members of the same group compared to the average distance to members of the nearest other group used in cluster analysis (Shahapure and Nicholas, 2020). We visualized and compared specimen representations with UMAP, a non-linear dimensionality reduction method that preserves local neighborhoods in a dataset but not absolute distances between points (McInnes et al., 2020).

We examined the activations of the final layer in the ResNet-18 encoder for each network to gain insights into the representation they learned. Each encoder produced specimen representations of 512 units and, therefore, had 512 channels. To limit the number of channels we looked at, we selected the channel from each network with the greatest standard deviation in representations of the training specimens. We produced images optimized to give the maximum and minimum activation for these channels (Olah et al., 2017). We also selected example specimens from the training dataset that produced a range of activations from the lowest to the highest.

### Applying Representations

We assessed how well the representations learned by the networks generalized by applying them to three potential applications.

#### Taxonomic Identification at Different Scales

Identification of herbarium specimens is a common taxonomic task at herbaria for both research and curation. Despite significant recent progress, practical details still need to be resolved before automated specimen identification is incorporated into day-to-day research and curation workflows. Generalized specimen representations may help resolve some of these details, potentially allowing researchers to build smaller, bespoke identification models that could be beneficial where limited computing resources are available.

During curation in herbaria, incoming specimens are often sorted at a higher taxonomic level before fine-scaled determination by an expert. Therefore, specimen representations should perform well across the taxonomic hierarchy, from order down to genus and, ideally, species.

##### Application Data

We used the three test sets split from the *Half*-*Earth* dataset (Table 2) to represent settings where the specimens are from herbaria not used to train the feature-extractor neural network (test set **H**), the specimens are for newly digitized species (test set **S**), and the specimens are from the same herbarium and species used to train the feature-extractor (test set **R**).

##### Application Method

We extracted representations of specimens for each of the three test sets using the three trained feature-extractor networks, resulting in nine groups of specimen representations. We then used multinomial logistic regressions to predict the order, family, and genus of a specimen for each group of representations, resulting in 27 models.

We measured the top-1 accuracy, macro-averaged precision, and macro-averaged F1-score of each model by fivefold cross-validation. We used L2 normalization to prevent overfitting and sample weighting to balance the classes in the datasets.

#### Discrimination of Similar and Distinct Genera

Often a researcher will want to know if a specimen belongs to one of two possible taxa rather than all possibilities. The differences between these two taxa may be fairly obvious, but the specimens need to be sorted quickly, or the differences may be difficult even for an expert to tell apart. Generalized specimen representations should allow successful discrimination in both cases. *Syzygium* and *Eugenia* are two closely related and visually similar genera in the family Myrtaceae that are often misidentified as each other, while *Dendrobium* is a large genus of orchids from Southeast Asia and is therefore easily distinguished from *Syzygium*.

##### Application Data

We downloaded all available images held at Royal Botanic Gardens, Kew for *Syzygium* (1,996), *Eugenia* (8,358), and *Dendrobium* (1,004) from iDigBio (Table 2). We resized all images to 256 × 256 pixels but did not subject them to any of the pre-processing steps of the *Half-Earth* dataset, like label blurring.

##### Application Method

After extracting representations for the three genera using the three feature-extractor networks, we trained one logistic regression model to distinguish between *Syzygium* and *Eugenia* (similar genera) and one to distinguish between *Syzygium* and *Dendrobium* (distinct genera) for each set of representations from the networks. We evaluated the accuracy and f1-score of the models using fivefold cross-validation and used L2 normalization and sample weighting, as before.

#### Identification of Mislabeled Specimens

Recent estimates suggest that over half of specimens for some plant groups may be mislabeled in digitized collections (Goodwin et al., 2015) due to genuine misidentifications, mistakes during digitization, or delays in updating names to the latest determination. However, identifying mislabeled specimens is difficult without expert taxonomic inspection of each image. Representations of specimen images could be used as the input for methods to identify such errors rapidly.

##### Application Data

We used the same representations of images for *Syzygium*, *Eugenia*, and *Dendrobium* as in the previous task. However, to simulate mislabeled specimens, we swapped the labels of 10% of specimens in each genus, between *Syzygium* and *Eugenia*, and *Syzygium* and *Dendrobium*.

##### Application Method

We predicted the probability of a specimen belonging to one of the two genera in each pair using an L2 penalized logistic regression, with representations extracted by each of our three neural networks as the inputs. To prevent over-confident predictions, we made these predictions on the validation fold of each split in a fivefold cross-validation. We then used the method implemented in *cleanlab* (Northcutt et al., 2021) for identifying the most likely mislabeled specimens based on the joint distribution of noisy and true labels in the data.

### Software

We used Python version 3.8 to perform all analyses in this study. We used *fastai* to load and transform the image data and *pytorch lightning* to build and train the models. We sub-classed the ResNet-18 autoencoder architecture from *pytorch lightning bolts*. We used *lucent*, a pytorch port of *lucid*, to visualize neural network channel activations. We used *scikit-learn* to build all linear models, implement cross-validation, and calculate silhouette scores.

## Results

### Learning Representations

**FIGURE 3:**
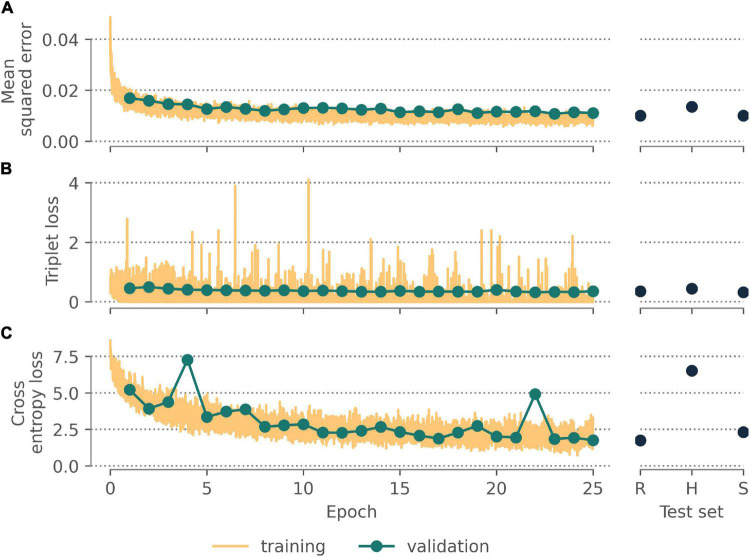
Performance of the **(A)** autoencoding neural network, **(B)** triplet network, and **(C)** classifier after training for 25 epochs on the validation data, a held-out test data set of randomly selected images, a held out test data set of all images for randomly selected species, a held out test data set of all images from institutions other than New York Botanic Gardens.

All neural networks appeared to converge after 25 epochs (Figure 3), with final validation losses of 0.011 (autoencoder, MSE), 0.358 (triplet network, triplet loss), and 1.758 (classifier, cross-entropy loss). These final networks achieved comparable losses on held-out test sets comprising randomly selected specimens (0.010; 0.354; 1.745) and specimens for unseen species (0.010; 0.323; 2.308) but higher losses for the held-out images from unseen herbaria (0.0136; 0.442; 6.520).

**FIGURE 4:**
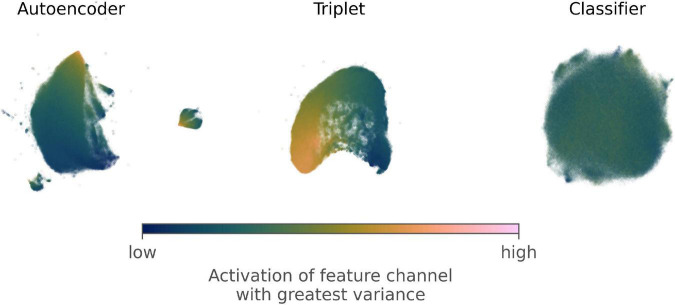
2-dimensional visualizations of feature vectors extracted from the training and validation images derived from the Half-Earth dataset by an autoencoding neural network, a triplet network, and a classifier. The 2-dimensional embeddings were generated using UMAP, a non-linear dimensionality reduction technique that aims to preserve that local neighborhoods in a dataset rather than absolute distances between points. Each visualization is colored by the relative value of the channel with the greatest standard deviation in the feature extraction layer of the corresponding network.

2-dimensional embeddings of the representations extracted by each network (Figure 4) appeared to show decreasing structure in the representations as supervision increased. Overlaying values of the channel with the greatest standard deviation from the feature extraction layer of each network also showed clear gradient in the embeddings from the autoencoder and triplet network but not for the embeddings from the classifier. However, the average silhouette score showed the opposite trend (Table 3), suggesting supervised learning increases the separability of classes at all levels of the taxonomic hierarchy. Overall, silhouette scores were negative, indicating a high degree of overlap for all taxonomic groupings.

**TABLE 3:** Silhouette scores for taxonomic groupings in representations of the Half-Earth dataset from three different neural networks.

Model	Genus	Family	Order
Autoencoder	−0.27	−0.19	−0.12
Triplet network	−0.38	−0.40	−0.30
Classifier	−0.14	−0.11	−0.05

**FIGURE 5:**
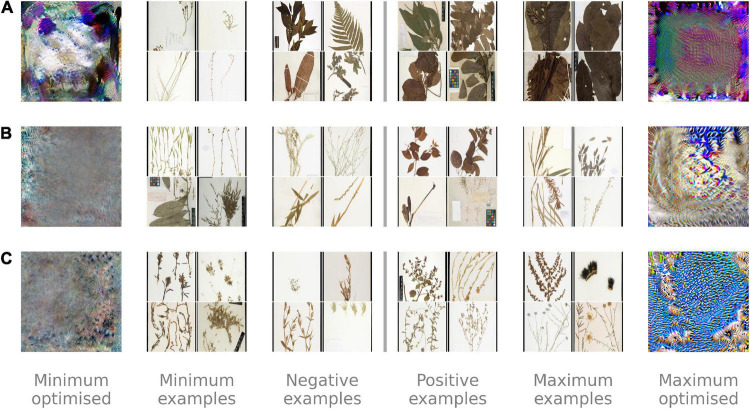
A visualization of the features that are extracted by the **(A)** autoencoding neural network, **(B)** triplet network, and **(C)** classification network after training on the Half-Earth dataset of herbarium specimen images. Only one out of the 512 channels in the extracted features are visualized for each network, chosen as the channel with the greatest standard deviation in the training dataset. We generated images optimized to produce the minimum (left) and maximum (right) output from these channels and selected example images from the training dataset that had the most negative, slightly negative, slightly positive, and most positive activations. Activations and examples for the autoencoder **(A)** suggest it is separating specimens based on how much of the image is covered, while the triplet network **(B)** and classification network **(C)** separate specimens using finer-scale details.

We visualized the optimized activations for the representation channel with the largest standard deviation for the training and validation images (Figure 5). These channels all appeared to pick up on textures across the images rather than well-defined features. Example images with contrasting high and low activations make it clear the channel with the greatest variation for the autoencoder was discriminating between light, thin specimens at low activation and dark, broad-leaved specimens at high activation. The activations for the triplet network and classifier were harder to interpret but may have separated images based on the presence of long blade-like sections and repeating feathery structures, respectively.

## Applying Representations

### Taxonomic Identification at Different Scales

**FIGURE 6:**
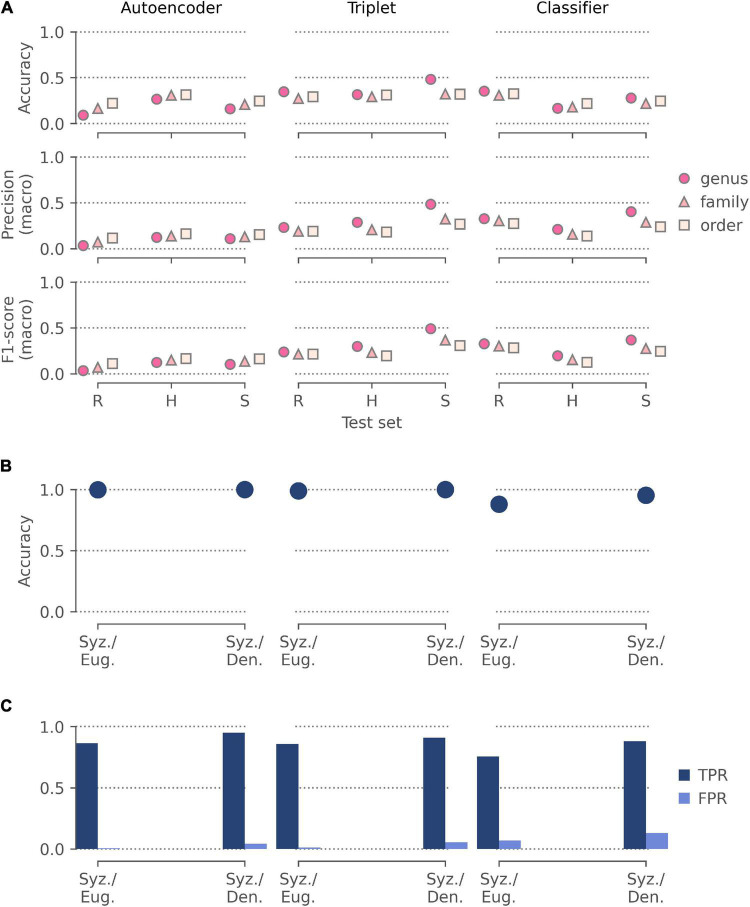
The performance of features extracted by our pre-trained autoencoder, triplet, and classifier networks in three applications: **(A)** identifying the order, family, and genus for the held-out test sets of random specimen images (R), images from unseen herbaria (H), and images of unseen species (S) from the Half-Earth dataset; **(B)** discriminating between specimens of two similar (*Syzygium* and *Eugenia*) and two distinct (*Syzygium* and *Dendrobium*) genera; **(C)** identifying mislabeled specimens in these two sets of specimens. All three applications used logistic regression models for classification and were evaluated by fivefold cross-validation using: **(A)** accuracy, macro-averaged precision, and macro-averaged F1-score; **(B)** accuracy. Application **(C)** used the cross-validated predictions to identify likely mislabeled specimens and was assessed using the proportion of mislabeled specimens correctly identified (true positive rate; TPR) and the proportion of correctly labeled specimens wrongly identified as mislabeled (false positive rate; FPR).

Models trained on the autoencoder representations were the most sensitive to the coarseness of the task, with accuracy improving from genus to order for all held out test sets (Figure 6A). Models trained on the triplet and classifier representations were less affected by the coarseness of the task, with their highest accuracies being achieved when predicting the genus of the held-out herbaria images (47.3%) and the randomly held-out specimen images (33.9%), respectively. Overall, the models trained on the triplet representations performed the best across all test sets.

### Discrimination of Similar and Distinct Genera

All models showed high accuracy when discriminating between two representations of two genera extracted from images from Kew’s herbarium (Figure 6B). The models trained on autoencoder and triplet representations achieved near-perfect accuracy for both the similar and distinct genera. However, the models trained with the classifier did not perform as well and showed higher accuracy discriminating between *Syzygium* and *Dendrobium* (95.3%) than between *Syzygium* and *Eugenia* (87.9%).

### Identification of Mislabeled Specimens

Similarly, models trained on the classifier representations correctly identified the fewest mislabeled specimens (Figure 6C) for both the similar (66.4%) and distinct genera (80.0%). Models trained on the autoencoder and triplet representations identified over 80% of mislabeled specimens in both sets of genera while incorrectly flagging fewer than 5% of correctly labeled specimens. The triplet representations were the best for identifying mislabeled specimens between similar genera (85.2%), while the autoencoder representations were slightly better with distinct genera (88.0%).

## Discussion

### Learning Representations

Our results highlight the differences between the representations learned by the three neural networks under different levels of supervision. Evaluating the separability of taxa in this representation space, we found that the Classifier produced the most separable representations. This result is perhaps expected, as supervised training aims to maximize the differences between the target classes. Perhaps more surprising, though, was that the representations produced by the Triplet network were the least separable.

Visualizations of the optimized activations for the channel from each network with the highest variation in the training data confirmed that the level of supervision influenced the information encoded in the representations. While the most active channel of the Autoencoder appeared to pick up on the amount of an image filled by the specimen, both the Triplet and Classifier networks discriminated between thin branching structures and repeating blob-like shapes. Despite the subjective interpretations that these visualizations necessitate, we found them vital for contrasting between the different networks and diagnosing any potential problems with the representations learned.

Although there was no indication from the activation visualizations that the neural networks were encoding spurious information, like the presence of a scale bar, in the representations, we did not investigate all channels of the feature extraction layer. With more investigation, the need to mask the specimen from the rest of the image may become apparent. We should be able to build on a recently published workflow which generates masks for specimens of ferns, though this will need evaluation to ensure that features seen in other vascular plant groups (such as flowers and fruits) are properly handled. A comprehensive masking strategy effective across vascular plant groups will allow us to determine the effect of masking specimens on the applications of their representations.

### Applying Representations

We have demonstrated that representations from all three networks generalize well to different classification tasks through our three application tasks. Although the accuracies achieved in the first application (taxonomic identification across scales) were all below 50%, this was the most challenging task. The best performing models, trained on representations extracted by the Triplet network, had a similar accuracy across all held-out test sets and at all levels of the taxonomic hierarchy. These results are encouraging for the prospect of using specimen representations to build lightweight models for identifying herbarium specimens where computational resources are limited.

The better performance in this task from representations extracted by the Classifier and Triplet networks over those from the Autoencoder aligns with the reported advantage of supervised over self-supervised methods for representation learning. However, the Autoencoder and Triplet representations achieved better results than those from the Classifier in the other two application tasks. Overall, the Triplet network gave the best results across all tasks, suggesting that although some supervision is beneficial, too much might overfit the representations to the task they were trained on.

### Improving Representations

Our study presents the first steps in applying representation learning to herbarium collections, and there is much we can try to improve these representations. While we used an autoencoding network as our example of self-supervision, contrastive methods like SimCLR (Chen et al., 2020a) offer an alternative that can approach the performance of supervised methods in some applications. However, these contrastive models can be expensive to train and, despite their promise, may not work well across all domains (Cole et al., 2021).

Recent work in the camera trap literature has generated improved representations from self-supervised learning by using context information about the time and location of images to develop spatio-temporal priors (Mac Aodha et al., 2019) and identify likely related images (Pantazis et al., 2021). Herbarium specimens are accompanied by rich context about their collection and identification histories and how they relate to each other through duplicates deposited across different herbaria and co-citation networks (Nicolson et al., 2018). As in the camera trap applications, this context could be used to improve representations learned by self-supervision. However, it could also be used to define novel methods for sampling triplets of images during training of a triplet network, as could information about the geographic or phylogenetic distance between specimens.

Specimen representations worked surprisingly well for discriminating between two genera and identifying mislabeled specimens. However, we need to evaluate their use across a broader range of tasks like counting organs on a specimen sheet, detecting phenology, and picking out low-quality specimens. As these are frequent tasks across different research projects and herbarium collections, we could usefully define a set of tasks as a resource to evaluate future approaches. The NeWT dataset for benchmarking representation learning using natural world images offers a template for achieving this goal (Van Horn et al., 2021).

## Conclusion

Our investigation has demonstrated the potential benefits of representation learning in the setting of herbarium collections. By contrasting different levels of supervision, we have identified metric learning through a triplet network as providing the best balance between fully and self-supervised representation learning. We evaluated the use of herbarium specimen representations across three tasks and found they were particularly effective for discriminating between genera and identifying mislabeled specimens. Although our representations achieved only mediocre performance in fine-grained taxonomic identification, we have identified several routes for improving the learning of herbarium specimen representations. Overall, we believe representation learning offers a way of harnessing large-scale digitized collections for the benefit of researchers working across all 3,500 herbaria worldwide. We intend to further investigate how we can use the rich context surrounding herbarium specimens to construct task sets and datasets to further develop this research area.

## Data Availability Statement

All images from the Half-Earth Challenge dataset are publicly available at https://www.kaggle.com/c/herbarium-2021-fgvc8. We have archived the RBG, Kew specimen images used in applications 2 and 3 at https://doi.org/10.5281/zenodo.5777843. The code used to carry out this study is archived at https://doi.org/10.5281/zenodo.5776894.

## Author Contributions

BW, AT, and NN contributed to the conception and design of the study. BW performed the analysis and wrote the first draft. All authors contributed to manuscript revision, read, and approved the submitted version.

## Conflict of Interest

The authors declare that the research was conducted in the absence of any commercial or financial relationships that could be construed as a potential conflict of interest.

## Publisher’s Note

All claims expressed in this article are solely those of the authors and do not necessarily represent those of their affiliated organizations, or those of the publisher, the editors and the reviewers. Any product that may be evaluated in this article, or claim that may be made by its manufacturer, is not guaranteed or endorsed by the publisher.
